# HbA1c reduction following flash monitoring commencement is not independently associated with adverse diabetic eye disease outcomes in type 1 diabetes

**DOI:** 10.1136/bmjdrc-2020-001668

**Published:** 2020-11-01

**Authors:** Muhammad Al-Dalla Ali, Roland H Stimson, Anna R Dover, Shareen Forbes, Roxanne Annoh, Karen Madill, Fraser W Gibb

**Affiliations:** 1 Edinburgh Centre for Endocrinology & Diabetes, Royal Infirmary of Edinburgh, Edinburgh, UK; 2 University/BHF Centre for Cardiovascular Science, University of Edinburgh, Edinburgh, UK; 3 Princess Alexandra Eye Pavilion, NHS Lothian, Edinburgh, UK

**Keywords:** diabetic retinopathy, glycated hemoglobin a, diabetes mellitus, type 1

## Abstract

**Introduction:**

Intensification of therapy has been associated with early worsening of retinopathy prior to subsequent risk reduction. We sought to assess whether glycated hemoglobin (HbA1c) reduction, following flash monitoring, was associated with early worsening.

**Research design and methods:**

An observational study in 541 individuals with type 1 diabetes and paired HbA1c and eye assessment prior to and following flash monitoring commencement.

**Results:**

Change in HbA1c was −4 mmol/mol (IQR −9–1) (−0.4% (−0.8–0.1)) and 25% achieved a fall in HbA1c of ≥10 mmol/mol. The occurrence of the composite end point (panretinal photocoagulation, macular laser or anti-VEGF therapy) was associated with baseline HbA1c >75 mmol/mol (9.0%) (HR 4.0 (95% CI 2.0 to 7.9), p<0.001) but not with fall in HbA1c of ≥10 mmol/mol (0.9%) (HR 1.6 (95% CI 0.8 to 3.2), p=0.203) over a follow-up period of 615 days (527–863). In multivariate analysis, diabetes duration (p=0.035) and prior retinopathy (p<0.001) were most predictive of the composite end point. Baseline HbA1c was the strongest predictor of worsening retinopathy (p=0.002) or new retinopathy (p=0.002) in multivariate analysis whereas change in HbA1c was not independently associated with either (p=0.930 and p=0.830, respectively).

**Conclusions:**

Progression of eye disease is associated with baseline HbA1c, diabetes duration and previous retinopathy and such individuals should be monitored during intensification of glycemic therapy. Reassuringly, the extent of glucose lowering does not appear to be an independent risk factor for early worsening of eye disease in this context.

**Significance of this study**

**What is already known about this subject?**

- Glycated hemoglobin (HbA1c) lowering has been associated with early worsening of diabetic retinopathy before risk reduction occurs.

**What are the new findings?**

- Baseline HbA1c and diabetes duration were associated with subsequent need for intervention for diabetic eye disease and with the development or worsening of diabetic eye disease.
- Change in HbA1c was not greater in those with subsequent panretinal photocoagulation, worsening of retinopathy or new development of retinopathy.
- When stratified based on HbA1c response, there were no differences in any eye outcomes when adjusted for baseline HbA1c.

**How might these results change the focus of research or clinical practice?**

- People with markedly elevated HbA1c, prior retinopathy and long diabetes duration require careful monitoring after commencement of flash monitoring, but these data offer reassurance that extent of HbA1c lowering may not be a major contributor to risk.

## Introduction

Flash glucose monitoring provides users with an interstitial glucose value only on scanning a glucose sensor with a reader device or compatible mobile phone. In other respects, it is similar to conventional continuous glucose monitoring (CGM) in providing a 24 hours glucose trace and trend arrows.^1^ We have previously shown that flash monitoring is associated with clinically important reduction in glycated hemoglobin (HbA1c) in type 1 diabetes, particularly in people with above target HbA1c at baseline.^2^ Flash monitoring is also known to reduce hypoglycemia and glucose variability in people with HbA1c ≤58 mmol/mol (7.5%) prior to commencement. Intensification of glycemic control reduces the long-term risk of microvascular complications in type 1 diabetes,^3^ although, in the Diabetes Control and Complications Trial (DCCT), an early worsening of diabetic retinopathy (EWDR) was observed before long-term, sustained risk reduction accrued.^4^ It is possible, however, that the method of intensifying glycemic control may influence the risk of early worsening of diabetic eye disease. An analysis of recent CGM randomized controlled trials suggests that intensification using CGM is associated with a significantly lower risk of hypoglycemia compared with self-monitored blood glucose (SMBG).^5^ Improved glycemic control, in the context of flash monitoring, is likely to be associated with lower rates of hypoglycemia and less glucose variability than was previously possible.^6^ Consequently, we hypothesized that improvement in glycemic control, following commencement of flash monitoring, may not independently predict early worsening of diabetic eye disease. To test this, we prospectively assessed the need for diabetic eye disease intervention (panretinal photocoagulation (PRP), macular laser or intravitreal antivascular endothelial growth factor (VEGF) therapy) or progression/development of diabetic eye disease in a cohort of individuals commencing flash monitoring, with particular reference to achieved fall in HbA1c.

## Participants and methods

### Study design and participants

We conducted a prospective observational study of the first 589 individuals with type 1 diabetes commenced on National Health Service (NHS) funded flash monitoring (Freestyle Libre, Abbott, Witney, UK) in a University hospital clinic (Royal Infirmary of Edinburgh) during February and March 2018. Glycemic outcomes for this cohort (including a group from another clinic within our center) have previously been reported, where the baseline characteristics are described in detail.^2^ In this current study, 14 individuals were excluded due to death or moving from the hospital catchment area, 14 were excluded due to absence of paired HbA1c data and a further 20 were excluded due to absence of paired eye outcome data, leaving a total cohort of 541 individuals (online supplemental figure). Two hundred twelve (39.2%) individuals had self-funded flash monitoring use prior to 2018 and provided their commencement date on a questionnaire. The study was entirely observational (with no deviation from standard clinical care) and ethics approval was not required.

10.1136/bmjdrc-2020-001668.supp1 Supplementary data

### Outcomes

The primary outcome was an assessment of factors associated with the development of a composite end point comprising PRP, macular laser therapy or anti-VEGF therapy. Additional outcomes of interest were the individual components of the composite outcome, new onset of retinopathy, new onset of maculopathy and worsening of retinopathy. Retinopathy was classified as: none, mild background, intermediate (any eye disease between mild background and proliferative, including all non-proliferative retinopathy and people with previous PRP and stable eye disease) or proliferative. Worsening retinopathy was defined as at least one step up through these categories, with the most advanced category being recorded for individuals when a discrepancy existed between eyes. Retinopathy and maculopathy data were obtained from the national diabetic retinopathy screening programme,^7^ which is accessible via our national clinic database system, SCI-Diabetes. In individuals attending specialist eye clinics, data on severity of eye disease and treatments administered were obtained from our hospital’s electronic health records. All individuals had a first eye assessment following flash monitoring with a further ‘final’ assessment available in 397/541 (73.4%) (online supplemental figure). Change in HbA1c was defined as the difference between HbA1c prior to commencement of any flash monitoring and the next available value after the flash monitoring education session (and change from baseline to the final available HbA1c). We also report the proportion of individuals achieving the Scottish HbA1c target (<58 mmol/mol (7.5%)). HbA1c was measured by ion-exchange high performance liquid chromatography using the Arkray Adams A1c automated platform (A. Menarini Diagnostics) and is typically measured every 6 months in people attending our clinics. Mode of insulin delivery (multiple daily injection (MDI) or continuous subcutaneous insulin infusion (CSII)), smoking status and urinary albumin status were obtained from SCI-Diabetes. Self-reported hypoglycemia data were available in 356/541 (65.8%) individuals from clinic questionnaires (including Gold score and a modification of the Clarke assessment^8^) completed within a year of commencing flash monitoring.

### Statistical analysis

Data were largely non-normally distributed (as determined by Shapiro-Wilk test) and are presented as median and IQR. Paired data were analyzed by Wilcoxon signed-rank test and unpaired data by Mann-Whitney U test. Categorical data were analyzed by χ^2^ test or by Fisher’s exact test, when assumptions for χ^2^ test were not met. Logistic regression analysis was performed to identify associations with development or worsening of eye disease. Univariate analysis of the composite end point and need for PRP were assessed by log-rank test. Independent predictors of the composite end point and need for PRP were assessed by Cox proportional-hazard model. Significance was accepted at p<0.05. All analyses were performed using R Studio V.1.0.153.

## Results

### Participant characteristics

Clinical and demographic characteristics are summarized in table 1. There was no significant difference between people achieving at least a 10 mmol/mol (0.9%) fall in HbA1c and those who did not, with the exception of baseline HbA1c which was significantly higher in responders (72 mmol/mol (65–83) vs 61 (53–67)) (8.7% (8.1–9.7) vs 7.7 (7.0–8.3), p<0.001).

**Table 1:** Clinical and demographic features of total cohort and comparing those achieving 10 mmol/mol (0.9%) or greater fall in HbA1c with those who did not

	Total cohort	HbA1c fell by 10 mmol/mol or greater	HbA1c did not fall by 10 mmol/mol or greater	P value
	N=541	N=135	N=406	
Male gender	277/541 (51.2%)	64/135 (47.4%)	213/406 (52.5%)	0.309
					Scottish index of multiple deprivation (SIMD) rank	4705 (2695–6399)	4746 (3245–6468)	4684 (2647–6369)	0.27
Age (years)	46 (34–57)	46 (34–55)	46 (34–58)	0.862
Age at diagnosis (years)	19 (11–31)	19 (12–29)	19 (11–31)	0.482
Baseline HbA1c (mmol/mol/%)	63 (55–72)/7.9 (7.2–8.7)	72 (65–83)/8.7 (8.1–9.7)	61 (53–67)/7.7 (7.0–8.3)	<0.001
Diabetes duration (years)	23 (13–34)	24 (14–32)	23 (13–34)	0.454
CSII	147 (27.2%)	33 (24.4%)	114 (28.1%)	0.411
Interval between flash monitoring start and next eye assessment (days)	235 (136–344)	259 (146–361)	225 (135–340)	0.081
Interval between flash monitoring start and final eye assessment (days)	615 (527–863)	660 (553–803)	601 (520–856)	0.109
Any retinopathy present at baseline	275 (50.8%)	72 (53.3%)	203 (50.0%)	0.502
Any maculopathy present at baseline	95 (17.6%)	30 (22.2%)	65 (16.0%)	0.1
Prior vitreous haemorrhage	14 (2.6%)	5 (3.7%)	9 (2.2%)	0.354
Prior vitrectomy	3 (0.6%)	2 (1.5%)	1 (0.2%)	0.155
PRP at baseline	57 (10.5%)	15 (11.1%)	42 (10.3%)	0.929
Macular laser at baseline	28 (5.2%)	8 (5.9%)	20 (4.9%)	0.65
Anti-VEGF at baseline	8 (1.5%)	3 (2.2%)	5 (1.2%)	0.419
Composite end point at baseline	70 (12.9%)	21 (15.6%)	49 (12.1%)	0.296
Smoking status	Current 52/501 (10.4%)	Current 12/126 (9.5%)	Current 40/375 (10.7%)	0.325
	Ex 126/501 (25.1%)	Ex 38/126 (30.2%)	Ex 88/375 (23.5%)	
	Never 323/501 (64.5%)	Never 76/126 (60.3%)	Never 247/375 (65.9%)	
Urinary albumin status	Macro 17/478 (3.6%)	Macro 6/122 (4.9%)	Macro 11/356 (3.1%)	0.238
	Micro 34/478 (7.1%)	Micro 12/122 (9.8%)	Micro 22/356 (6.2%)	
	None 427/478 (89.3%)	None 104/122 (85.2%)	None 323/356 (90.7%)	

P value refers to comparison between HbA1c response groups.

CSII, continuous subcutaneous insulin infusion; HbA1c, glycated hemoglobin; PRP, panretinal photocoagulation; VEGF, vascular endothelial growth factor.

### HbA1c and hypoglycemia

At baseline, 31.4% had an HbA1c <58 mmol/mol (7.5%) which rose to 48.6% at next follow-up after commencing flash monitoring (p<0.001). Median change in HbA1c was −4 mmol/mol (−9–1) (−0.4% (−0.8–0.1)) at next follow-up (median 253 days (156–507), p<0.001) and −3 mmol/mol (−8–4) (−0.3% (−0.7–0.4)) at final follow-up (median 703 days (603–888), p<0.001). HbA1c reduction was more marked in individuals with HbA1c >75 mmol/mol (9.0%) at baseline (−11 mmol/mol (−23–−5), p<0.001) (−1.0% (−2.1–−0.5)). At baseline, 31.4% had an HbA1c <58 mmol/mol (7.5%), which rose to 48.6% at next follow-up after commencing flash monitoring (p<0.001); 47.5% achieved an HbA1c fall of 5 mmol/mol (0.5%) or greater (sustained until final follow-up in 62.9%), 25.0% achieved a fall of 10 mmol/mol (0.9%) (sustained until final follow-up in 54.5%) and 8.1% achieved a fall of 20 mmol/mol (1.8%) (sustained until final follow-up in 52.5%). Self-reported hypoglycemia measures, following commencement of flash monitoring, were not significantly different in those achieving an HbA1c reduction of 10 mmol/mol (0.9%) and those who did not (online supplemental table 1).

### Changes in diabetic eye disease

Median follow-up for occurrence of the composite end point (PRP, macular laser or anti-VEGF therapy) was 615 days (527–863) from commencement of flash monitoring. During this time, 34 of 541 individuals experienced an event at a median 218 days (112–469) from flash monitoring commencement, of whom 26 had a previous element of the composite end point prior to commencement (23 PRP, 10 macular laser, 6 anti-VEGF). Twenty-seven individuals required PRP following flash monitoring commencement (median 187 days (100–382)), which was the first ever episode in 7/27 (details presented in table 2).

**Table 2:** Clinical features of individuals with first episode of PRP occurring after commencement of flash monitoring

	Age range (years)	Diabetes duration (years)	Baseline HbA1c (mmol/mol/%)	Smoking status	Urine albumin status	HbA1c change at next follow-up (mmol/mol/%)	Severe hypoglycemia in previous year	HbA1c change at final follow-up	MDI/CSII	Baseline retinopathy status	Days flash monitoring prior to PRP	Final retinopathy status	Days flash monitoring to final retinopathy status
1	30–40	8	64/8.0	NA	NA	−6/−0.6	No	NA	CSII	Proliferative	155	Mild.background	1241
2	30–40	14	83/9.7	Never	Normal	+4/+0.4	NA	−10/−0.9	MDI	None	635	Proliferative	678
3	30–40	6	80/9.5	Ex	Normal	−24/−2.2	No	−31/−2.8	MDI	Mild background	387	Non-proliferative	927
4	50–60	32	66/8.2	Current	Normal	−8/−0.7	No	NA	MD	Non-proliferative	1076	Proliferative	1076
5	40–50	21	57/7.4	Ex	Normal	−2/−0.2	NA	+12/1.1	MDI	Mild non-proliferative	767	Proliferative	965
6	60–70	8	84/9.8	Ex	Normal	−22/−2.0	NA	0/0	MDI	Mild background	165	NA	NA
7	30–40	14	82/9.7	NA	NA	−10/−0.9	NA	0/0	MDI	Mild background	284	Proliferative	1228

CSII, continuous subcutaneous insulin infusion; HbA1c, glycated hemoglobin; MDI, multiple daily injection; NA, not available; PRP, panretinal photocoagulation.

Three people received macular laser after flash monitoring all of whom had received previous macular laser for maculopathy previously. Nine people received anti-VEGF therapy after flash monitoring (median 222 days (186–542)), all of whom had maculopathy diagnosed prior to commencement and eight of whom had received previous laser treatment.

At next follow-up, since commencement of flash monitoring (median 235 days (135–344)), 430 (79.5%) had no change in their retinopathy status (none, mild background retinopathy, intermediate or proliferative). A deterioration was observed in 59 (10.9%) and improvement in 52 (9.6%). Next follow-up was within 6 months in 209/541 (47.3%) and within 12 months in 442 (81.7%). By final follow-up (n=397, median 615 days (527–863)), the corresponding figures were: no change in 279 (70.3%), deterioration in 72 (18.1%) and improvement in 46 (11.6%). For maculopathy, at first assessment, no change was observed in 496 (91.7%), deterioration in 28 (5.2%) and improvement in 17 (3.1%). The corresponding maculopathy figures at final assessment were 388 (97.7%), 3 (0.8%) and 6 (1.5%), respectively. Two hundred sixty-six individuals had no retinopathy at baseline of whom 48 (18.0%) had developed retinopathy at next follow-up (all mild background).

### Univariate analysis: associations with diabetic eye disease

#### Composite end point

**Figure 1:**
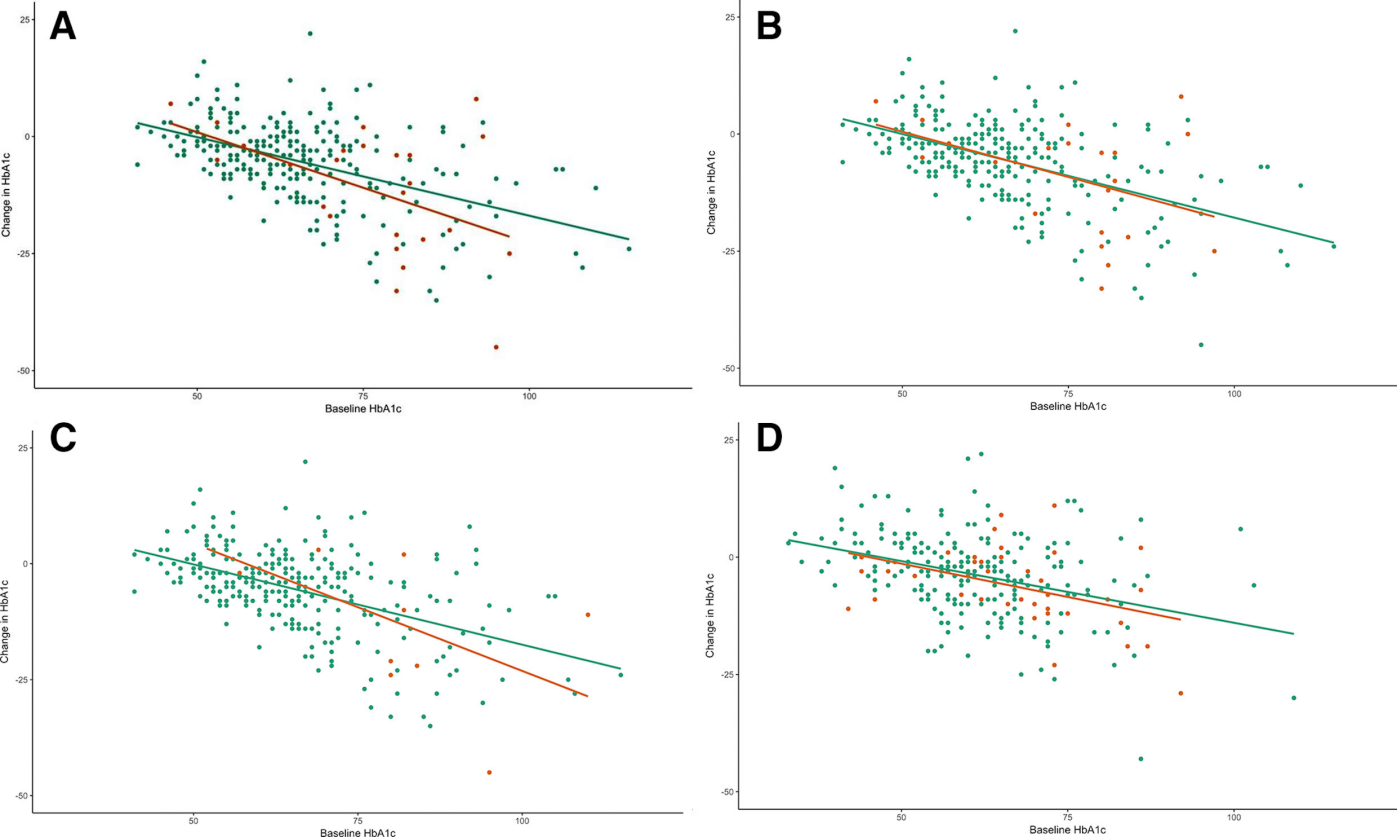
Association between baseline glycated hemoglobin (HbA1c) and change in HbA1c following flash monitoring. (A) Composite end point in cohort with retinopathy at baseline. (B) Individuals requiring panretinal photocoagulation (PRP) in cohort with retinopathy at baseline. (C) Individuals with worsening retinopathy status in cohort with retinopathy at baseline. (D) Onset of new retinopathy in cohort with no pre-existing retinopathy. Orange dots represent events and green dots indicate individuals with no event.

**Figure 2:**
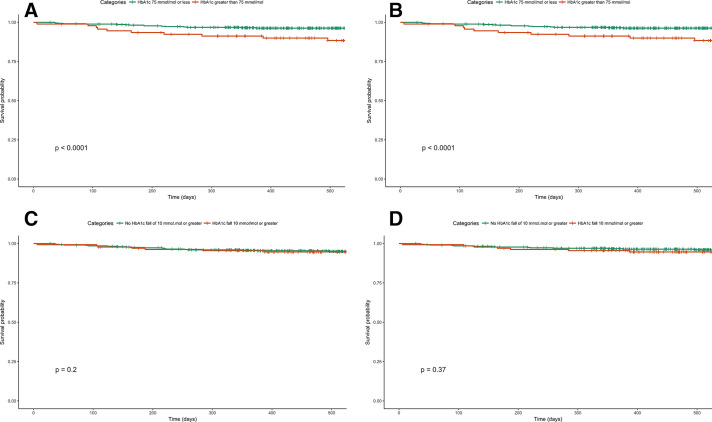
Survival curves stratified by baseline glycated hemoglobin (HbA1c) category (≤75 mmol/mol vs >75 mmol/mol). (A) Composite end point. (B) Panretinal photocoagulation (PRP). Survival curves stratified by baseline fall in HbA1c category (10 mmol/mol or >10 mmol/mol) vs <10 mmol/mol). (C) Composite end point. (D) PRP. Vertical lines indicate censored data.

Younger age at diagnosis (13 years (8–21) vs 19 (12–31), p=0.010), longer diabetes duration (27 years (24–38) vs 23 (12–36), p=0.001), higher HbA1c at baseline (74 mmol/mol (57–81) vs 63 (55–71), p=0.006) (8.9% (7.4–9.6) vs 7.9 (7.2–8.6)) and the presence of previous eye disease or treatments (table 3) were all associated with occurrence of the composite end point following flash monitoring. Change in HbA1c from baseline was not different between those requiring an intervention and those who did not (−5 mmol/mol (−17–−1) vs −4 (−9–1), p=0.163) (−0.5% (−1.6–−0.1) vs −0.4 (−0.8–0.1)) (table 3 and figure 1A). The HR for the composite end point in those with high baseline HbA1c (>75 mmol/mol (9.0%)) was 4.0 (95% CI 2.0 to 7.9, p<0.001) (figure 2A), but there was no significant association in those with HbA1c fall ≥10 mmol/mol (0.9%) (HR 1.6 (95% CI 0.8 to 3.2), p=0.203) (figure 2C).

**Table 3:** Univariate comparison of clinical features by occurrence of composite end point, PRP, worsening retinopathy and new-onset retinopathy

	Composite end point	No composite end point	P value	PRP	No PRP	P value	Worsening retinopathy	No worsening retinopathy	P value	New retinopathy	No new retinopathy	P value
	N=34	N=507		N=27	N=514		N=59	N=482		N=48	N=218	
Male gender	22/34 (64.7%)	255/507 (50.3%)	0.104	18/27 (66.7%)	259/514 (50.4%)	0.099	33/59 (55.9%)	244/482 (50.6%)	0.441	25/48 (52.1%)	106/218 (48.6%)	0.664
Age (years)	45 (35–57)	46 (34–57)	0.501	44 (34–56)	46 (34–58)	0.873	48 (35–60)	46 (34–57)	0.342	47 (35–59)	45 (30–58)	0.403
Age at diagnosis (years)	13 (8–21)	19 (12–31)	0.01	13 (8–21)	19 (12–22)	0.004	18 (12–31)	19 (11–30)	0.946	19 (12–31)	23 (14–37)	0.195
Diabetes duration (years)	27 (24–38)	23 (12–36)	0.001	27 (22–36)	23 (13–34)	0.009	25 (15–35)	23 (13–34)	0.302	24 (14–33)	14 (8–30)	<0.001
Baseline HbA1c (mmol/mol/%)	74 (57–81)	63 (55–71)	0.006	75 (59–82)	63 (55–71)	0.005	69 (48–81)	63 (55–71)	0.01	65 (57–73)	61 (54–68)	0.014
	8.9 (7.4–9.6)	7.9 (7.2–8.6)		9.0 (7.5–9.7)	7.9 (7.2–8.6)		8.5 (6.5–9.6)	7.9 (7.2–8.6)		8.1 (7.4–8.8)	7.7 (7.1–8.4)	
Change in HbA1c (mmol/mol/%)	−5 (-17–-1)	−4 (−9–1)	0.163	−4 (−15–0)	−4 (−9–1)	0.548	−4 (−12–0)	−4 (−9–1)	0.226	−4 (−7–0)	−3 (−9–2)	0.176
	−0.5 (−1.6–0.1)	−0.4 (−0.8–0.1)		−0.4 (−1.4–0)	−0.4 (−0.8–0.1)		−0.4 (−1.1–0)	−0.4 (-0.8–0.1)		−0.4 (−0.6–0)	−0.3 (−0.8–0.2)	
HbA1c fall 10 mmol/mol (0.9%)	12/34 (35.3%)	123/507 (24.3%)	0.15	9/27 (33.3%)	126/514 (24.5%)	0.302	21/59 (35.6%)	114/482 (23.7%)	0.045	15/63 (23.8%)	48/218 (22.0%)	0.173
CSII	12/34 (35.3%)	135/507 (26.6%)	0.271	10/27 (37.0%)	137/514 (26.7%)	0.237	17/42 (28.8%)	130/482 (27.0%)	0.763	16/48 (33.3%)	57/218 (26.1%)	0.312
Baseline retinopathy	30/34 (88.2%)	245/507 (48.3)	<0.001	25/27 (92.6%)	250/514 (48.6%)	<0.001	11/59 (18.6%)	264/482 (54.8%)	<0.001	NA	NA	NA
Baseline maculopathy	25/34 (73.5%)	70/507 (13.8%)	<0.001	18/27 (66.7%)	77/514 (15.0%)	<0.001	10/59 (16.9%)	85/482 (17.6%)	0.896	NA	NA	NA
PRP at baseline	23/34 (67.6%)	34/507 (6.7%)	<0.001	20/27 (74.1%)	37/514 (7.2%)	<0.001	4/59 (6.8%)	53/482 (11.0%)	0.319	NA	NA	NA
Macular laser at baseline	10/34 (29.4%)	18/507 (3.6%)	<0.001	5/27 (18.5%)	23/514 (4.5%)	0.001	4/59 (6.8%)	24/482 (5.0%)	0.781	NA	NA	NA
Anti-VEGF at baseline	6/34 (17.6%)	2/507 (0.4%)	<0.001	3/27 (11.1%)	5/514 (1.0%)	<0.001	2/59 (3.4%)	6/482 (1.2%)	0.473	NA	NA	NA
Composite at baseline	26/34 (76.5%)	44/507 (8.7%)	<0.001	20/27 (74.1%)	50/514 (9.7%)	<0.001	8/59 (13.6%)	62/482 (12.9%)	0.88	NA	NA	NA
Smoking status	C 3/29 (10.3%)	C 49/472 (10.4%)	0.991	C 3/23 (13.0%)	C 49/478 (10.3%)	0.87	C 1/55 (1.8%)	C 51/446 (11.4%)	0.084	C 1/45 (2.2%)	C 19/200 (9.5%)	0.264
	E 7/29 (24.1%)	E 119/472 (25.2%)		E 5/23 (21.7%)	E 121/478 (25.3%)		E 16/55 (29.1%)	E 110/446 (24.7%)		E 13/45 (28.9%)	E 50/200 (25.0%)	
	N 19/29 (65.5%)	N 304/472 (64.4%)		N 15/23 (65.2%)	N 308/478 (64.4%)		N 38/55 (69.1%)	N 285/446 (63.9%)		N 31 (68.9%)	N 131/200 (65.5%)	
Albumin status	Macro 0/28 (0.0%)	Macro 17/450 (3.8%)	0.783	Macro 0/22 (0.0%)	Macro 17/456 (3.7%)	0.568	Macro 5/51 (9.8%)	Macro 12/427 (2.8%)	0.037	Macro 8/192 (4.2%)	Macro 5/41 (12.2%)	0.126
	Micro 2/28 (6.1%)	Micro 32/450 (7.1%)		Micro 1/22 (4.5%)	Micro 33/456 (7.2%)		Micro 4/51 (8.2%)	Micro 30/427 (7.0%)		Micro 11/192 (5.7%)	Micro 2/41 (4.9%)	
	None 26/28 (92.9%)	None 401/450 (89.1%)		None 21/22 (95.5%)	None 406/456 (89.0%)		None 42/51 (82.4%)	None 385/427 (90.2%)		None 173/192 (90.1%)	None 34/41 (82.9%)	

C, current; CSII, continuous subcutaneous insulin infusion; E, ex-smoker; HbA1c, glycated hemoglobin; HbA1c, glycated hemoglobin; N, never smoked; PRP, panretinal photocoagulation; VEGF, vascular endothelial growth factor.

#### Panretinal photocoagulation

Younger age at diagnosis (13 years^8–21^ vs 19 (12–22), p=0.004), longer diabetes duration (27 years (22–36) vs 23 (13–34), p=0.009), higher HbA1c at baseline (75 mmol/mol (59–82) vs 63 (55–71), p=0.005) (9.0% (7.5–9.7) vs 7.9% (7.2–8.6)) and the presence of previous eye disease or treatments (table 3) are all associated with the need for PRP following flash monitoring. Change in HbA1c from baseline was not different between those requiring PRP and those who did not (−4 mmol/mol (−15–0) vs −4 (−9–1), p=0.548) (−0.4% (−1.4–0.0) vs −0.4 (−0.8–0.1)) (table 3 and figure 1B). The HR for the composite end point in those with high baseline HbA1c (>75 mmol/mol (9.0%)) was 4.7 (95% CI 2.1 to 10.0, p<0.001) (figure 2B) but there was no significant association in those with HbA1c fall ≥10 mmol/mol (0.9%) (HR 1.4 (95% CI 0.6 to 3.2), p=0.368) (figure 2D).

#### Worsening retinopathy

Worsening retinopathy status at next follow-up was associated with higher baseline HbA1c (69 mmol/mol (48–81) vs 63 (55–71), p=0.010) (8.5% (6.5–9.6) vs 7.9 (7.2–8.6)) and lower frequency of retinopathy at baseline (18.6% vs 54.8%, p<0.001) at first follow-up. It was not significantly associated with change in HbA1c following flash monitoring commencement (table 3 and figure 1C). At final follow-up, diabetes duration (26 years (17–37) vs 23 (12–34), p=0.028) and lower frequency of retinopathy at baseline (22.2% vs 54.5%, p<0.001) were associated with worsening retinopathy (online supplemental table 2). The OR for the worsening retinopathy, at first follow-up, was 2.2 (p=0.012) in those with baseline HbA1c >75 mmol/mol (9.0%) and 1.8 (p=0.045) in those with HbA1c fall ≥10 mmol/mol (0.9%).

#### New-onset retinopathy

New onset of retinopathy (all of which was mild background) was associated with longer diabetes duration (24 years (14–33) vs 14 (8–30), p<0.001) and higher baseline HbA1c (65 mmol/mol (57–73) vs 61 (54–68), p=0.014) (8.1% (7.4–8.8) vs 7.7 (7.1–8.4)) but not change in HbA1c (−4 mmol/mol (−7–0) vs −3 (−9–−2), p=0.176) (−0.4 (−0.6–0) vs −0.3 (−0.8–−0.2)) table 3 and figure 1D). No other parameters were significantly associated with new onset of retinopathy (table 3). At final follow-up, diabetes duration (25 years (15–37) vs 14 (8–29), p<0.001) and younger age at diagnosis (15 years (10–26) vs 26 (15–38), p<0.001) were associated with worsening retinopathy (online supplemental table 2). The OR for the new-onset retinopathy, at first follow-up, was 2.2 (p=0.065) in those with HbA1c >75 mmol/mol (9.0%) at baseline and was 1.6 (p=0.173) for those with fall ≥10 mmol/mol (0.9%).

### Multivariate analysis: associations with diabetic eye disease

Cox proportional-hazards analysis identified diabetes duration (HR 1.03 (95% CI 1.00 to 1.06) per year, p=0.035) and presence of baseline retinopathy (HR 6.58 (95% CI 2.26 to 19.20), p<0.001) but not baseline HbA1c (HR 1.03 (95% CI 1.00 to 1.05), p=0.075) or change in HbA1c (HR 0.99 (95% CI 0.96 to 1.03), p=0.702) as independently predictive of the composite end point.

Cox proportional-hazards analysis identified baseline HbA1c (HR 1.03 per mmol/mol (95% CI 1.01 to 1.06), p=0.015) and presence of baseline retinopathy (HR 11.5 (95% CI 2.64 to 50.2), p=0.001) but not change in HbA1c (HR 1.01 per mmol/mol (95% CI 0.98 to 1.05), p=0.568) or diabetes duration (HR 1.02 per year (95% CI 0.99 to 1.05), p=0.153) as independently predictive of subsequent PRP.

HbA1c at baseline (OR 1.04 per mmol/mol (95% CI 1.01 to 1.06), p=0.002) and diabetes duration (OR 1.03 per year (95% CI 1.01 to 1.05), p=0.005) but not change in HbA1c (OR 1.00 per mmol/mol (95% CI 0.97 to 1.04), p=0.830) were independently predictive of new development of retinopathy in logistic regression analysis. HbA1c at baseline (OR 1.03 per mmol/mol (95% CI 1.01 to 1.05), p=0.002) but not diabetes duration (OR 1.01 per year (95% CI 0.99 to 1.03), p=0.261) or change in HbA1c (OR 1.00 per mmol/mol (95% CI 0.97 to 1.03), p=0.930) were independently predictive of worsening retinopathy in logistic regression analysis.

## Discussion

We have demonstrated that HbA1c lowering, in the context of flash monitoring commencement, is not independently predictive of clinically important changes in diabetic eye disease in the short-term. HbA1c prior to commencement of flash monitoring, as well as diabetes duration and pre-existing retinopathy were all independently associated with progression of eye disease. Intensification of glycemic management in type 1 diabetes is unequivocally associated with reduction in the development and progression of diabetic retinopathy,^3 9 10^ but those randomized to intensive glycemic control in the DCCT experienced EWDR prior to accruing substantial and sustained benefit in the longer term.^4^ A number of potential pathophysiological mechanism have been posited to explain this phenomenon, including perturbations of the somatotropic axis, increased retinal concentration of VEGF and changes in other angiogenic growth factors.^11^ However, the evidence in support of these proposed mechanisms is, at best, mixed.

We hypothesized that advances in the management of type 1 diabetes since the early 1980s (eg, modern CSII, insulin analogues, aggressive blood pressure management and ACE inhibitor prescribing) and specific features of intensification relating to flash monitor use may have reduced the risk of early worsening of eye disease following HbA1c reduction. Randomization to intensive glycemic control in DCCT was associated with a threefold increase in severe hypoglycemia while we did not, as previously reported, observe an increase in severe hypoglycemia following commencement of flash monitoring.^2^ RCT evidence attests to reduction in hypoglycemia (and glucose variability) during flash monitoring use^6^ in individuals with on-target HbA1c at baseline and similar findings have emerged from a large French observational study with respect to severe hypoglycemia.^12^ Analysis of data from two recent real-time CGM studies, in MDI users, has suggested a significant attenuation of the increased risk of hypoglycemia as HbA1c levels fall, in contrast to those using SMBG.^5^ glycemic variability is another potential contributor towards risk of diabetes complications^13^ and has been associated with structural damage to the neuroretina in type 1 diabetes based on CGM measures of variability,^14^ while visit-to-visit variability in HbA1c has been independently associated with retinopathy progression in adolescents with type 1 diabetes.^15^ Lending support to the potential importance of reduced glucose variability and hypoglycemia, early worsening of retinopathy has not been reported in the context of HbA1c reduction following islet^16 17^ and pancreas transplantation.^18^ Islet transplantation is associated with both reduced rates of hypoglycemia and lower glucose variability.^19^ Similarly, no significant progression of retinopathy was observed in a study of people with type 1 diabetes following CSII commencement.^20^

The cohort described in our study is different from the DCCT cohort in a number of important respects: older age (46 vs 27 years), longer duration of diabetes (23 vs 6 years) and higher prevalence of prior diabetic eye disease intervention (12.9% vs none in DCCT). Baseline HbA1c (63 mmol/mol (7.9%) vs 76 mmol/mol (9.1%)) was lower in our study, although baseline HbA1c in those with a 10 mmol/mol (0.9%) or greater fall in HbA1c (72 mmol/mol (8.7%)) was similar to the DCCT cohort. Our cohort is broadly representative of typical clinical practice in the UK, although, as previously described, it is slightly skewed toward lower than average HbA1c, younger age, CSII use and lower socioeconomic deprivation than our total clinic population.^2^

The study is open to the usual criticisms of observational methodology particularly the potential influence of unmeasured confounders. As a ‘real-world’ assessment, the timing of HbA1c measurement and eye assessment was not uniform and reflected the expected variation in normal clinical practice. A minority of individuals did not have an eye assessment within 1 year of flash monitoring commencement, meaning some early worsening may have been missed. Lack of uniformly timed HbA1c and eye assessments limits our ability to comment on the potential influence of rate of change in HbA1c. However, we have no reason to suspect variation in follow-up intervals introduced any systematic bias. An advantage of this ‘real-world’ methodology is its likely generalizability, in the absence of stringent exclusion and inclusion criteria, to modern diabetes clinic populations in the UK and beyond. The DCCT^3^ represents a landmark in methodologically robust evidence gathering in type 1 diabetes but the treatment options and risk factor management for the condition have evolved substantially since the 1980s and, to our knowledge, our study represents the largest assessment of factors associated with early worsening of eye disease in the context of modern intensification of glycemic control. A significant limitation of our study is the absence of detailed Gold Standard evaluation of retinopathy, typically considered to be the Early Treatment Diabetic Retinopathy Study (ETDRS) classification system.^21^ We relied on clinical data from our national screening programme^7^ and electronic health record entries from specialist eye clinic evaluation, which do not provide the same level of granularity as the ETDRS classification system. It is conceivable, therefore, that we have under-reported subtle changes in retinopathy. However, by relying on clinical data, we feel it is unlikely that we have missed clinically important changes and have also reported unambiguous hard end points (PRP, macular laser and anti-VEGF therapy). It is possible that we have missed an association between HbA1c lowering and EWDR because the degree of HbA1c reduction observed in this cohort is below the threshold at which this effect occurs, although this seems unlikely as none of the reported multivariate analyses of HbA1c reduction came close to approaching statistical significance. However, these data cannot exclude the possibility that more extreme reduction in HbA1c increases the risk of EWDR. It could also be argued that our results reflect a type 1 error, in refuting the independent association between glucose lowering and early EWDR, due to insufficient cohort size or events. However, in multivariate analysis of the composite end point, new development of retinopathy and worsening of retinopathy, the absence of association was clear, consistent and not close to approaching statistical significance. The study benefits from comprehensive follow-up data and a wide range of associated clinical parameters derived from our national diabetes database, however, we were not able to report information on ACE inhibitor prescribing (and other antihypertensives) and pregnancy which would have been of interest in the context of diabetic eye disease.

## Conclusions

Elevated HbA1c, longer duration of diabetes and pre-existing retinopathy are all significant risk factors with respect to diabetic eye disease in people with type 1 diabetes commencing flash monitoring. However, in the short-term, subsequent change in HbA1c does not appear to independently predict the risk of retinopathy development or progression nor the need for interventions to treat advanced eye disease in a representative cohort of flash monitoring users. These single-centre observational findings, while offering a degree of reassurance, clearly require corroboration from larger national datasets and randomized controlled trials of novel glucose-lowering technologies. Understanding the risk of abrupt reduction in HbA1c, as well as the potential moderating influence of hypoglycemia and glucose variability, will become increasingly important as we approach the era of closed-loop insulin delivery.
