# Supplementary material for: HbA1c reduction following flash monitoring commencement is not independently associated with adverse diabetic eye disease outcomes in type 1 diabetes
Source: BMJ Open Diabetes Res Care. 2020 Nov 1;8(2):e001668. doi: 10.1136/bmjdrc-2020-001668 (PMC7607593; doi:10.1136/bmjdrc-2020-001668)
Supplement: Supplementary data [file bmjdrc-2020-001668supp001.pdf]

Supplementary figure. Flow chart detailing missing data and follow-up intervals.

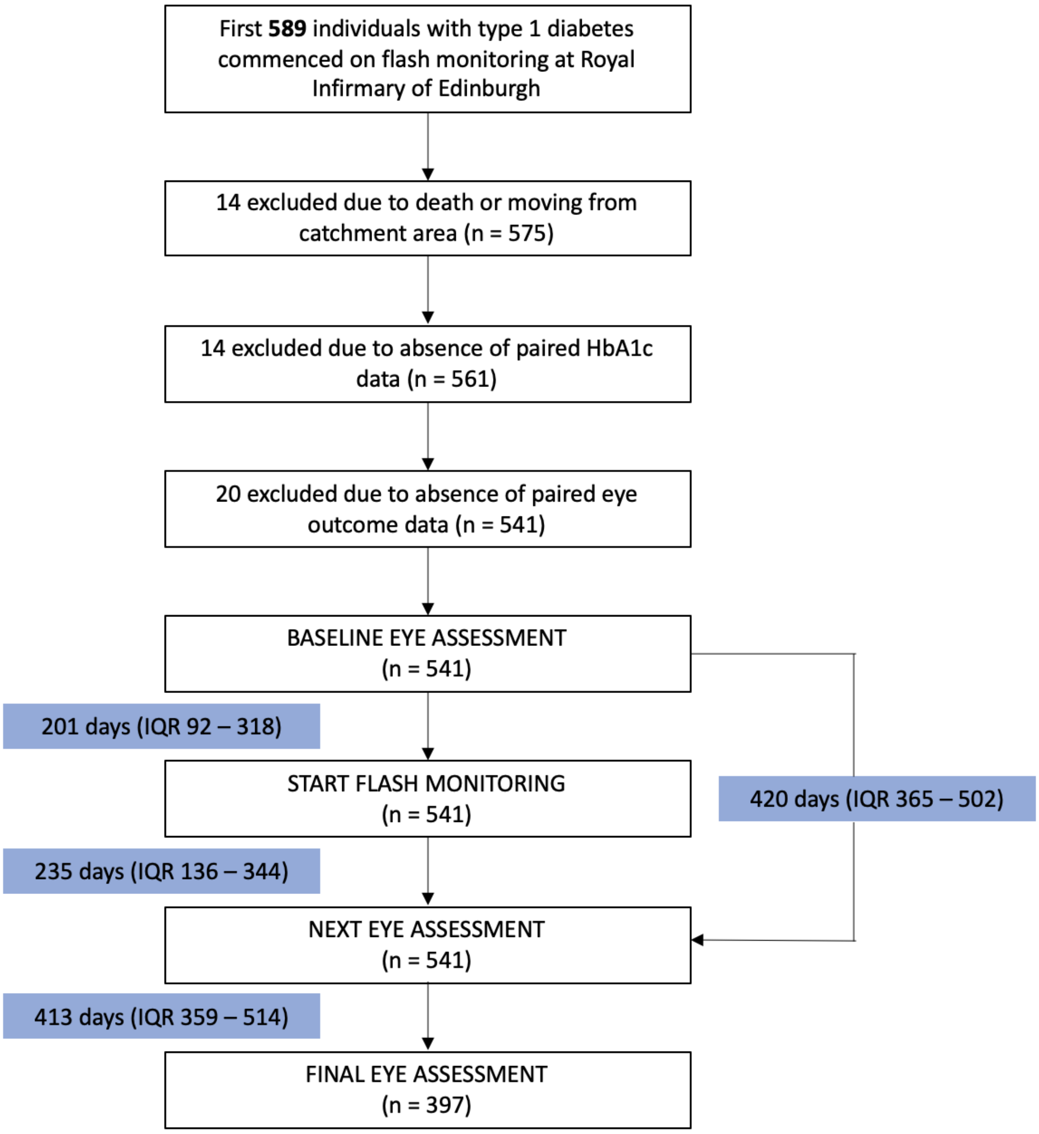

Supplementary Table 1. HbA1c and hypoglycaemia data following flash monitoring commencement stratified by HbA1c response. p refers to comparison between HbA1c response groups. Optimal bolus timing refers to people taking bolus insulin at least 15 minutes prior to meals.

	Total cohort N = 541	HbA1c fall 10 mmol/mol (0.9%) or greater N = 135	HbA1c change less than -10mmol/mol (0.9%) N = 406	p
Baseline HbA1c (mmol/mol / %)	63 (55 – 72) / 7.9 (7.2 – 8.7)	72 (65 – 83) / 8.7 (8.1 – 9.7)	61 (53 – 67) / 7.7 (7.0 – 8.3)	<0.001
Change in HbA1c (mmol/mol / %)	-4 (-9 – 1) / 0.4 (-0.8 – 0.1)	-16 (-21 – -12) / 1.5 (-1.9 – -1.1)	-2 (-5 – 2) / 0.2 (-0.5 – 0.2)	<0.001
Interval baseline to next HbA1c (days)	384 (260 – 608)	399 (286 – 607)	376 (252 – 606)	0.533
Optimal bolus timing	126/350 (36.0%)	40/89 (44.9%)	86/261 (33.0%)	0.042
Severe hypo last year?	40/356 (11.2%)	13/89 (14.6%)	27/267 (10.1%)	0.245
Symptomatic hypo more than weekly	159/347 (45.8%)	36/89 (40.4%)	123/258 (47.4%)	0.238
Asymptomatic hypo frequency once / month or more	101/348 (29.0%)	26/87 (29.9%)	75/261 (28.7%)	0.838
Impaired awareness hypoglycaemia?	45/347 (13.0%)	14/88 (15.9%)	31/259 (12.0%)	0.342

Supplementary Table 2. Univariate comparison of clinical features by worsening retinopathy and new-onset retinopathy at final follow-up assessment.

	Worsening retinopathy	No worsening retinopathy	p	New retinopathy	No new retinopathy	p
Male gender	36/72 (50.0%)	174/325 (53.5%)	0.586	27/58 (46.6%)	72/146 (49.3%)	0.721
Age (years)	46 (35 – 62)	46 (34 – 57)	0.585	46 (34 – 60)	47 (32 – 59)	0.983
Age at diagnosis (years)	15 (10 – 25)	19 (11 – 31)	0.103	15 (10 – 26)	26 (15 – 38)	<0.001
Diabetes duration (years)	26 (17 – 37)	23 (12 – 34)	0.028	25 (15 – 37)	14 (8 – 29)	<0.001
Baseline HbA1c (mmol/mol / %)	64 (54 – 74)<br>8.0 (7.1 – 8.9)	63 (56 – 70)<br>7.9 (7.3 – 8.6)	0.684	62 (52 – 73)<br>7.8 (6.9 – 8.8)	61 (55 – 68)<br>7.7 (7.2 – 8.4)	0.527
Change in HbA1c (mmol/mol / %)	-5 (-12 – 1)<br>-0.5 (-1.1 – 0.1)	-4 (-9 – 0)<br>-0.4 (-0.8 – 0)	0.384	-4 (-12 – 1)<br>-0.4 (-1.1 – 0.1)	-3 (-9 – 1)<br>-0.3 (-0.8 – 0.1)	0.609
HbA1c fall 10mmol/mol (0.9%)	23/72 (31.9%)	80/325 (24.6%)	0.199	17/58 (29.3%)	36/146 (24.7%)	0.494
CSII	22/72 (30.6%)	93/325 (28.6%)	0.743	17/58 (29.3%)	41/146 (28.1%)	0.861
Any retinopathy present at baseline	16/72 (22.2%)	177/325 (54.5%)	<0.001	NA	NA	NA
Any maculopathy present at baseline	16/72 (22.2%)	54/325 (16.6%)	0.259	NA	NA	NA
PRP at baseline	9/72 (12.5%)	39/325 (12.0%)	0.906	NA	NA	NA
Macular laser at baseline	8/72 (11.1%)	17/325 (5.2%)	0.063	NA	NA	NA
Anti-VEGF at baseline	3/72 (4.2%)	5/325 (1.5%)	0.331	NA	NA	NA
Composite at baseline	15/72 (20.8%)	44/325 (13.5%)	0.115	NA	NA	NA
Smoking status	Current 6/65 (9.2%)<br>Ex 17/65 (26.2%)<br>Never 42/65 (64.6%)	Current 35/302 (11.6%)<br>Ex 71/302 (23.5%)<br>Never 196/302 (64.9%)	0.809	Current 4/52 (7.7%)<br>Ex 11/52 (21.2%)<br>Never 37/52 (71.2%)	12/137 (8.8%)<br>37/137 (27.0%)<br>88/137 (64.2%)	0.660
Albumin status	Macro 5/60 (8.3%)<br>Micro 4/60 (6.7%)<br>None 51/60 (85.0%)	Macro 9/292 (3.1%)<br>Micro 25/292 (8.6%)<br>None 258/292 (88.4%)	0.154	Macro 4/48 (8.3%)<br>Micro 1/48 (2.1%)<br>None 43/48 (89.6%)	Macro 7/132 (5.3%)<br>Micro 11/132 (8.3%)<br>None 114/132 (86.4%)	0.268
